# Wettability of Nanostructured Transition-Metal Oxide (Al_2_O_3_, CeO_2_, and AlCeO_3_) Powder Surfaces

**DOI:** 10.3390/ma15165485

**Published:** 2022-08-10

**Authors:** Muidh Alheshibri, H. M. Albetran, B. H. Abdelrahman, A. Al-Yaseri, N. Yekeen, I. M. Low

**Affiliations:** 1 Department of Basic Sciences, Deanship of Preparatory Year and Supporting Studies, Imam Abdulrahman Bin Faisal University, P.O. Box 1982, Dammam 31441, Saudi Arabia; 2 Basic and Applied Scientific Research Center (BASRC), Imam Abdulrahman Bin Faisal University, P.O. Box 1982, Dammam 31441, Saudi Arabia; 3 Department of Physics, College of Science, Imam Abdulrahman Bin Faisal University, P.O. Box 1982, Dammam 31441, Saudi Arabia; 4 Department of Basic Sciences, College of Education, Imam Abdulrahman Bin Faisal University, Dammam 31451, Saudi Arabia; 5 Center of Integrative Petroleum Research (CIPR), College of Petroleum Engineering, King Fahd University of Petroleum and Minerals, Dhahran 31261, Saudi Arabia; 6 Department of Petroleum Engineering, Universiti Teknologi PETRONAS, Seri Iskandar 32610, Perak, Malaysia; 7 Department of Physics and Astronomy, Curtin University, GPO Box U1987, Perth, WA 6845, Australia

**Keywords:** wettability, transition-metal oxide, nanopowders, Al_2_O_3_, CeO_2_, AlCeO_3_

## Abstract

Wettability has been the focal point of many studies in metal oxide materials due to their applications in water–gas shift reactions, organic reactions, thermochemical water splitting, and photocatalysis. This paper presents the results of systematic experimental studies on the wettability of surfaces of nanostructured transition-metal oxides (TMOs) (Al_2_O_3_, CeO_2_, and AlCeO_3_). The wettability of nanoparticles was investigated by measuring contact angles of different concentrations of water-based nanofluids (0.05–0.1 wt%) on the glass slide. The morphology, the heterostructure, and the nature of incorporated nanoparticles were confirmed by scanning electron microscopy (SEM) and transmission electron microscopy (TEM). Characteristic diffraction patterns of the nanomaterials were evaluated using energy-dispersive X-ray spectroscopy (EDS) and X-ray diffraction (XRD) techniques. The contact angles of water–Al_2_O_3_, water–CeO_2_, and water–AlCeO_3_ were measured as 77.5 ± 5°, 89.8 ± 4°, and 69.2 ± 1°, respectively. This study suggests that AlCeO_3_ is strongly water-wet (hydrophilic), while CeO_2_ is weakly water-wet (hydrophobic). It further demonstrated that the sizes and compositions of the nanoparticles are key parameters that influence their wetting behaviors.

## 1. Introduction

The wettability of fine powders has been investigated in different industrial fields, including mineral and metallurgical, pharmaceutical, and agrochemical industries, as well as food and cosmetic industries, to enhance their physical and material properties, such as the mineral composition [1,2,3], separation selectivity [4], plastic recycling [5], oil recovery from reservoir rocks [6], and stability of aqueous-oil emulsions [7,8,9].

The wetting behavior of oil or water through the powders is therefore an essential condition to enhance their efficiency in different applications. Wettability, in general, describes the tendency of a surface to be wetted by liquid, and it is typically identified in terms of contact angle (θ), which is the angle between the tangent to the surface and the tangent to the liquid droplets at the air–liquid–solid interface. The contact angle is a crucial characteristic property that needs to be precisely measured between particulate solids and oil or water interfaces to indicate whether the powders are hydrophobic or hydrophilic.

The techniques used in the literature to determine the wettability of powders can be divided into two main categories: direct and indirect methods. The former is based on direct measurements of the solid–liquid interface, such as the sessile drop method, while the latter is based on indirect measurement of the apparent contact angle, such as the capillary rise test, inverse gas chromatography, and equilibrium height method. The sessile drop method is commonly used in determining the wettability of powders due its simplicity and availability even though the method is commonly applied to smooth and flat surfaces [10]. In this technique, the three-phase static and dynamic contact angles are measured using a contact angle goniometer. This method sometimes requires the high-pressure compression of powders into pellets. Another generally accepted use of the sessile drop method is for the measurement of the contact angles between the water and the powder of interest where the powder is deposited on a substate using adhesive tape [11,12,13,14].

Several studies have investigated the wetting properties of pharmaceutical powders such as salicylic acid and lactose powders with water, PEG, and PVC solutions [12,15]. On the contrary, less attention has been focused on the wettability of metal oxide powders even though they are extensively used in different applications such as the fabrication of welding wires, rods, electrodes, and powder metallurgy.

Iveson et al. [16] measured the wettability of iron ore powders, and the contact angle was found to be in the range of 30–70°. Furthermore, they observed an increase in the contact angles of the iron ores as the ratio of oxides increased. Nowak et al. [11] carried out a comprehensive study where they compared the wettability of metal oxide powders such as SiO_2_, Al_2_O_3_, TiO_2_, and ZrO_2_ on water and paraffin oil. TiO_2_ was found to be largely wettable in paraffin oil, unlike the SiO_2_, Al_2_O_3_, and ZrO_2_ powders, in which no noticeable change in their wettability was observed. However, a significant difference was observed in terms of the wettability of water, where the contact angles were found to be 91°, 75°, 132°, and 49° for Al_2_O_3_, SiO_2_, TiO_2_, and ZrO_2_, respectively.

Common metal oxides such as aluminum oxide (Al_2_O_3_), cerium oxide (CeO_2_), titanium oxide (TiO_2_), and zirconia (ZrO_2_) are commonly used in manufacturing ceramic materials due to their excellent heat and chemical resistance compared to other materials [17]. However, the surfaces of materials used in manufacturing porous ceramic membranes are naturally hydrophilic due to the presence of large quantities of hydroxyl groups. Thus, the wide applications of ceramic membranes are hindered by the wettability selectivity in ceramic materials [18]. Furthermore, metal oxide nanopowders, such as aluminum oxide nanoparticles, are commonly used in biomedical applications such as cancer therapy, biosensing, drug delivery, and cell targeting [19]. The interactions of these materials inside the blood stream are significantly dependent on their wettability properties. Cerium oxide materials have also attracted tremendous attention in the catalytic systems for a variety of applications, including the oxygen storage component in car converters, the water–gas shift reaction, organic reactions, thermochemical water splitting, and photocatalysis [20]. Al_2_O_3_ and CeO_2_ nanoparticles have been involved in a wide range of applications due to their low-cost manufacturing and easy handling. The combination of these two metal oxide nanoparticles has been used as a suitable sintering additive for the liquid-phase sintering of solid-state sintered ceramics [21]. Proper wettability in the composite makes them feasible for the liquid-phase sintering. The number of research papers dealing with the wettability of individual metal oxide powders and their mixtures is scarce. The wettability of TMO powders plays a great role in several applications, such as dispersions, coatings, as a precursor to dissolution, powder processing such as granulation, and other practical usages [10,22]. The wettability of transition-metal oxide surfaces is not well-understood because of the large variation in contact angle measurements and limited experimental data. The measurement of contact angles of powders is notoriously difficult due to the fact that different techniques can produce highly variable results. The contact angle measurements are influenced by many factors (e.g., solid surface roughness, temperature, relative humidity, surface contamination, sample pretreatment, and surface preparation [14]). However, the particle morphology of nanoscale transition-metal oxides can provide a large specific surface area, which is very sensitive to the contact angle and wettability.

In this study, the wettability of nanostructured transition-metal oxide powders (Al_2_O_3_, CeO_2_, and AlCeO_3_) was examined using contact angle measurements. The materials were characterized for particle size and composition by scanning electron microscopy (SEM), associated energy-dispersive X-ray spectroscopy (EDS), transmission electron microscopy (TEM), X-ray diffraction (XRD), and zeta-potential measurements.

## 2. Experimental Methodology

### 2.1. Materials and Instrumentations

Aluminum oxide nanoparticles (Al_2_O_3_), suspension, *Mw* = 101.96 g/mol), cerium oxide nanopowder (CeO_2_), *Mw* = 172.11 g/mol), and aluminum–cerium oxide nanopowders (AlCeO_3_), *Mw* = 215.10 g/mol) were purchased from Sigma-Aldrich. The Al_2_O_3_ was provided as a colloidal solution, and thus they were heated up to 200 °C prior to their use. Moreover, the nanopowders (NPs) were scattered using ultrasonic vibration for ~5 min before taking the measurements of contact angles.

The transition-metal oxide nanopowders were characterized using an X-ray diffractometer (XRD) (Shimadzu-7000, S/N Q30545200239sz, Japan) with Cu Kα radiation, operating at 30 mA, 40 kV, a sampling pitch of 0.2°, and at a 2°/min-scanning rate in the diffraction range of 2θ = 20° to 80°, and using scanning electron microscopy (SEM) (Tescan VEGA 3, Czech Republic), energy-dispersive X-ray spectroscopy (EDX) (Element Silicon Drift Detector AMETEK MATERIALS ANALYSIS DIVISION, USA), and transmission electron microscopy (TEM) (FEI, Morgagni, 268) to ascertain the morphologies, sizes, microstructures, and phase purities.

### 2.2. Measurements of Contact Angle of the Nanopowders

**Figure 1:**
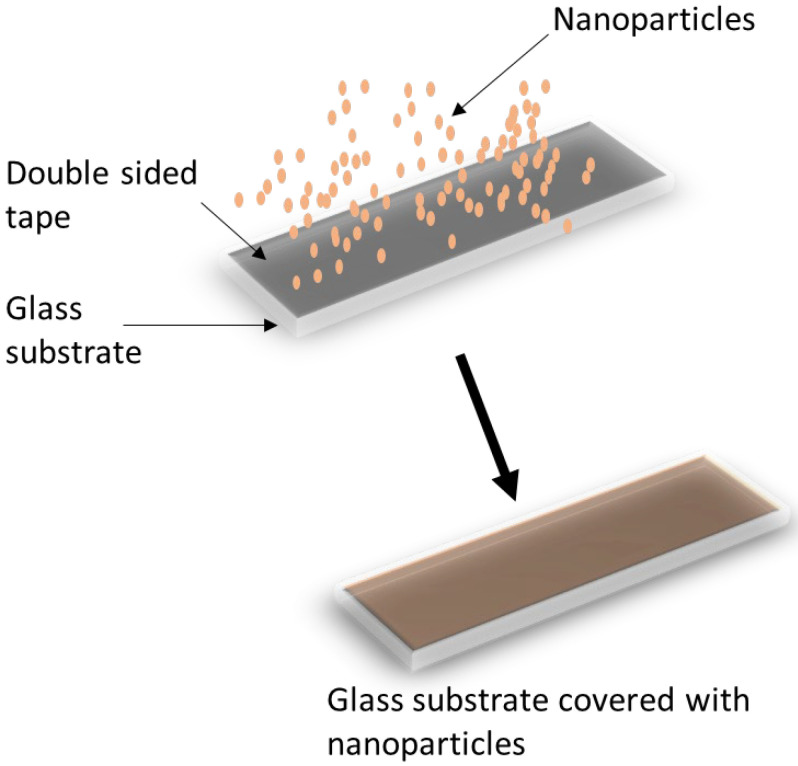
A schematic diagram of the prepared films used in this study.

A contact angle goniometer (Rame-Hart Instrument Co., Model 250, DROPimage Advanced software, USA) was utilized to measure the contact angles of water droplets on the nanopowders. The contact angles were measured using the glass slide method explained by Nowak et al. [11] and Shang et al. [14]. A schematic diagram is shown in Figure 1. Nowak et al. [11] showed that this method has minimal effect on the contact angle measurements. Besides its simplicity and availability, this technique has the advantage of studying the powder properties in their existing conditions. Furthermore, it is widely used for comparing the wettabilities of nanopowder materials. The nanopowders were placed on microscope slide covers (2 cm × 2 cm × 1.2 mm) with a double-sided adhesive tape. After 60 s, powders that were not rigidly adherent to the double-sided tape were omitted. Measurements were taken for the drop contact angle, which was stable within a 60 s timeframe. The volume of the water droplets used in this study was 4 µL. Each measurement recorded here was based on the average of three measurements at room temperature.

### 2.3. Zeta-Potential Measurements

Three nano-suspension fluids were prepared by preparing a suspension of 0.05–0.1 wt% Al_2_O_3_, CeO_2_, and AlCeO_3_ nanopowders, respectively, in MilliQ water. Each nanosuspension was sonicated for 15 min prior to measurements and then injected into the measurement cell. Zeta-potential measurements were conducted using a Malvern ZetaNano sizer (Zetasizer Nano ZS). For all measurements, 13 runs of 10 s were performed with at least five repetitions to obtain the average zeta potential.

## 3. Results and Discussion

### 3.1. Imaging of Microstructures

**Figure 2:**
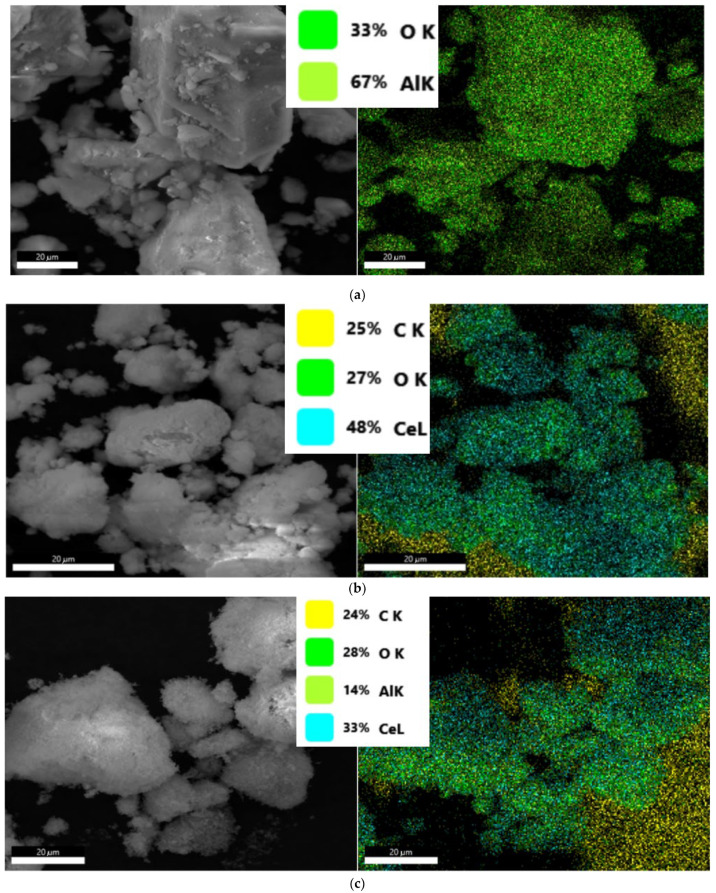
SEM micrographs and EDS of (**a**) Al_2_O_3_, (**b**) CeO_2_, and (**c**) AlCeO_3_ nanopowders.

The material particle sizes, morphologies, and microstructures of nanostructured transition-metal oxides (TMOs) (Al_2_O_3_, CeO_2_, and AlCeO_3_) were characterized by SEM and TEM. Images in Figure 2a–c show a typical secondary electron microscopy with a rough, cloudy-like texture, and a quantitative EDS mapping of the Al_2_O_3_, CeO_2_, and AlCeO_3_, respectively. The EDS mappings of the TMO samples show (33% O k and 67% Al k for Al_2_O_3_), (25% C k, 27% O k, and 48% Ce k for CeO_2_), and (24% C k, 28% O k, 14% Al K, and 33%, Ce K for AlCeO_3_). The C content in the CeO_2_, and AlCeO_3_ can be attributed to carbon coating to avoid any electric charging on the cerium material.

**Figure 3:**
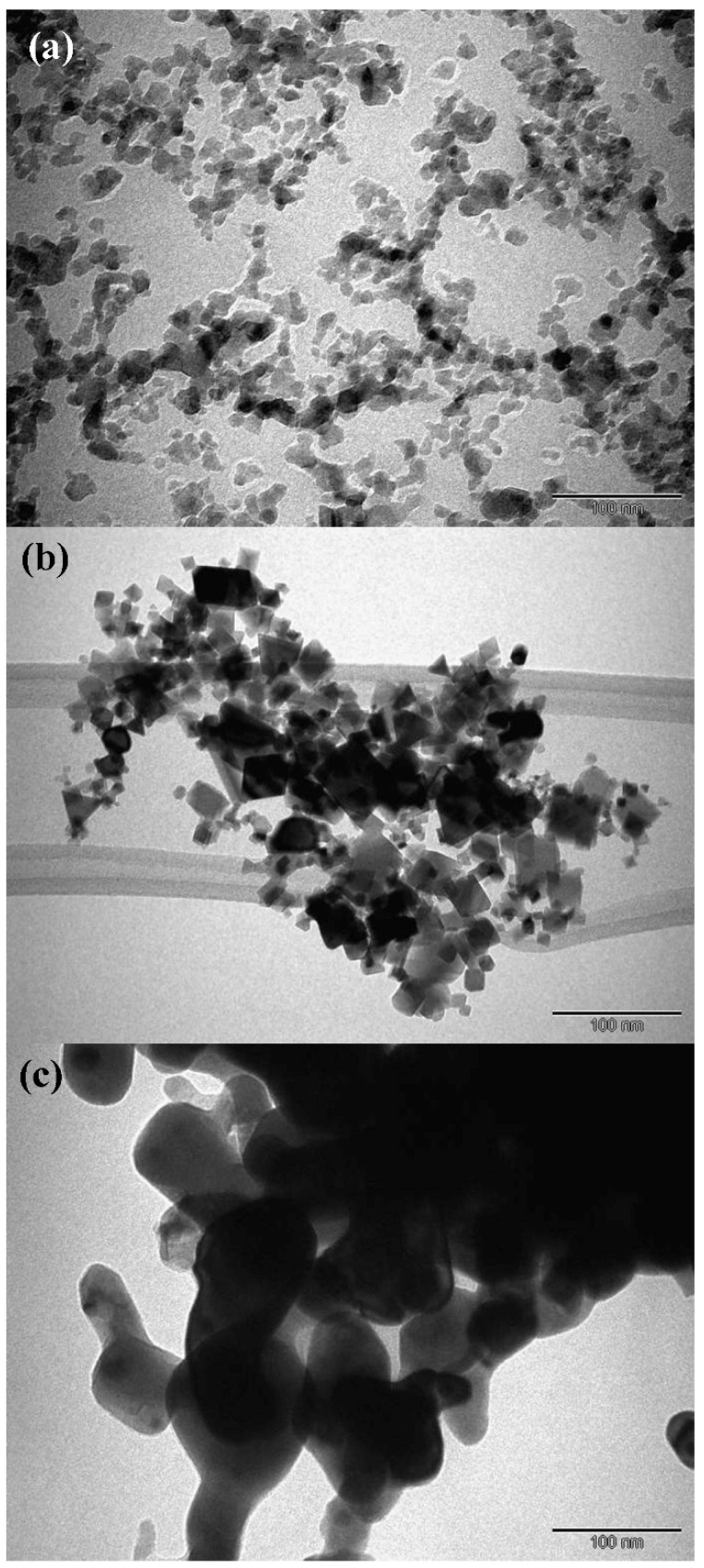
TEM micrographs of (**a**) Al_2_O_3_, (**b**) CeO_2_, and (**c**) AlCeO_3_ nanopowders.

The samples imaged by transmission electron microscopy for the Al_2_O_3_, CeO_2_, and AlCeO_3_ are shown in Figure 3a–c, respectively. The TEM images confirm the formation of the nanopowders with ≈10–80 nm nanoparticle sizes. Furthermore, it reveals nearly spherical or spheroid-shaped nanoparticles. These particles were found to be in agglomerated conditions. The sizes of the nanoparticles were measured using ImageJ^®^ software (version 1.48e) developed by the National Institutes of Health (NIH), Bethesda, MD, USA. The corresponding mean particles and standard deviation for 50 nanoparticles were 8 ± 2 nm, 16.6 ± 8 nm, and 56 ± 19 nm for Al_2_O_3_, CeO_2_, and AlCeO_3_, respectively.

### 3.2. XRD Analysis

**Figure 4:**
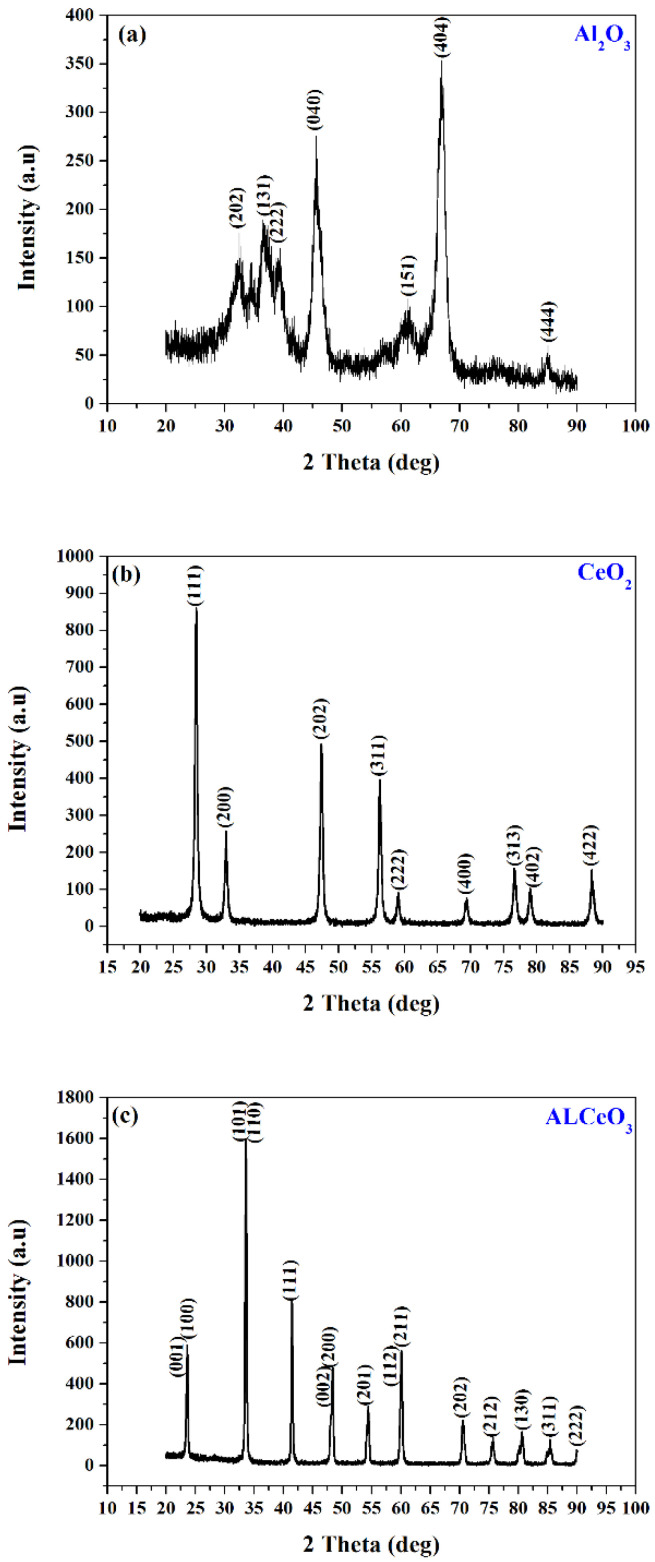
XRD patterns of (**a**) Al_2_O_3_, (**b**) CeO_2_, and (**c**) AlCeO_3_ nanopowders.

Plots in Figure 4a–c show the XRD patterns of nanostructured transition-metal oxides (Al_2_O_3_, CeO_2_, and AlCeO_3_), which were obtained from the COD-Inorganics reference database (COD-96-120-0016), (COD-96-900-9009), and (COD-96-152-5371), respectively. No impurities and second-phase peaks were observed in the crystalline TMOs.

The Scherrer equation was used to calculate the average crystallite size (*D_XRD_*) of TMOs [23,24,25]:

(1) $$D_{XRD}=\frac{k\lambda }{\beta cos\theta }$$

where *k*, *λ*, *β*, and *θ* are shape factor (0.91), wavelength of the Cu-K_α_ radiation (0.15419 nm), full width at half maximum (FWHM), and the Bragg diffraction angle, respectively.

The average TMO crystallite sizes (*D_XRD_*) of the Al_2_O_3_, CeO_2_, and AlCeO_3_ were 7, 21, and 30 nm, respectively. These size values are comparable to the average particle diameters from the TEM images.

### 3.3. Contact Angle Measurements

Contact angle measurements can be conducted using different techniques that are based on the geometry of solid materials. For powdered samples, contact angles are obtained using the wicking method [11,26]. It measures the speed of the capillary rise in the porous media, which can be used to determine the contact angles. However, this technique is hindered by the clustering and shrinking of the powders or soil colloids.

To prevent this problem, contact angles are measured using the glass slide method described by Nowak et al. [11] and Shang et al. [14], in which powders or porous materials are deposited onto a flat glass substrate, forming a layer of the powders or porous materials. This technique allows for studying the powder’s properties in its present condition. Furthermore, it is widely used for comparing the wettabilities of nanopowder materials.

Prior to contact angle measurements between the nanopowders and water, control measurements were taken to determine the contact angles between the water and both the clean slide and the slide covered with double-sided tape. The surface tension of water was 72.8 (mL/m^2^), and the contact angle between the water droplets and clean glass slide was found to be 5°. The contact angle between the water droplets and adhesive tape was 110°.

**Figure 5:**
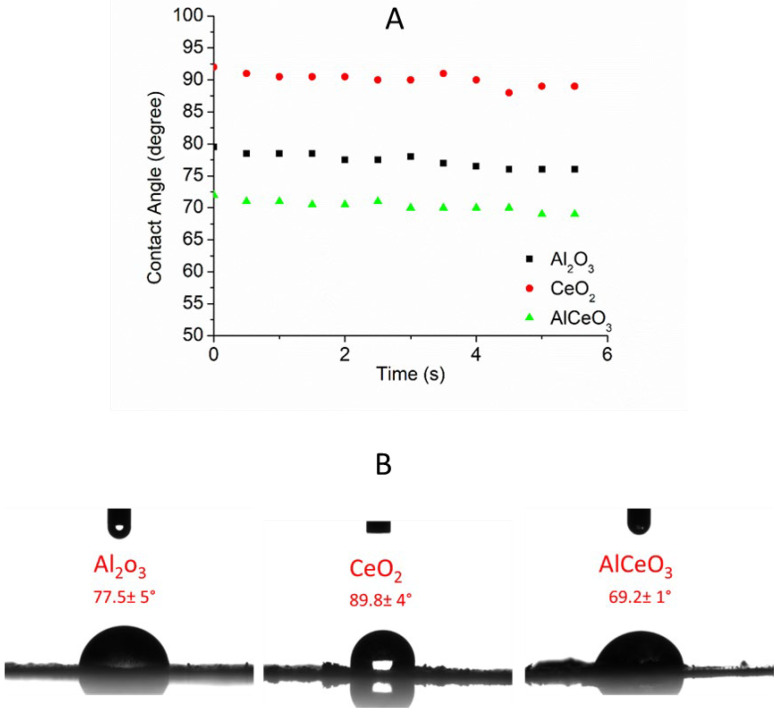
Contact angles of water droplets on Al_2_O_3_, CeO_2_, and AlCeO_3_ nanopowders determined with the static sessile drop method during the first 6 s (**A**) and images of water droplets and Al_2_O_3_, CeO_2_, and AlCeO_3_ nanopowders attached to a microscope glass slide with adhesive tape (**B**).

Figure 5a shows the contact angles as a function of time when the water droplets were placed onto the nanopowders. The contact angle between the water droplet and the glass covered with nanoparticles was measured immediately from the image of drops shown in Figure 5. The nanoparticles covered the surface completely as the contact angles were very different to the contact angles between the surface covered by adhesive tape. Furthermore, the contact angles between the water and nanopowders varied for each type of nanopowder. The contact angles were measured as 77.5 ± 5°, 89.8 ± 4°, and 69.2 ± 1° for the Al_2_O_3_, CeO_2_, and AlCeO_3_ nanopowders, respectively. Some might argue that the thickness of the metal oxide powder placed on the glass slide would affect the contact angles. This was unlikely to occur in this study since the different powder films were prepared in the same conditions, and powders that were not rigidly adherent to the double-sided tape were removed. Furthermore, it has been reported by Tavana that the thickness of prepared films does not affect the contact angle measurements [27]. They measured the contact angles of three different solvents on both dip-coated and spin-coated films with different thicknesses and did not observe any noticeable differences.

The results shown in Figure 5 clearly show that the contact angles with water were greatly affected by the chemical composition of nanoparticles. There was an approximately 12° difference between the contact angles obtained with Al_2_O_3_ and CeO_2_ nanoparticles. Surprisingly, water barely wet CeO_2_ nanoparticles, but it wetted its mixture with Al_2_O_3_ (i.e., θ_CeO2_ > θ_AlCeO3_). This difference is likely due to the size of nanoparticles (see Figure 2) and the aggregates observed in the Al_2_O_3_ nanopowders. Munshi et al. [28] showed that the contact angles depend on the size of nanoparticles for water and diethylene glycol droplets. The contact angles were shown to increase with an increase in the particle size. The experimental results were supported by a theoretical analysis. This is similar to the finding in this study where a lower contact angle was observed for small-sized nanoparticles (i.e., Al_2_O_3_ nanopowder), and a larger contact angle was observed for large-sized nanoparticles (i.e., CeO_2_ nanopowder). However, the contact angle for the AlCeO_3_ nanopowder did not follow the trend here. This is likely due to the larger polydispersity of the nanopowder given the fact that the AlCeO_3_ nanopowder was a mixture of both Al_2_O_3_ and CeO_2_ nanopowders. This led to an increase in the roughness of the powder sample, which eventually decreased the contact angles for hydrophilic surfaces. Minakov et al. [29] discussed the influence of nanoparticles on the oil wettability of different types of hydrophilic and hydrophobic rocks. They linked the variation in the contact angles to the size of the nanoparticles and showed that the contact angle increased with a decrease in the size of nanoparticles. This is the opposite of what was observed in this study. However, the comparison here is not conclusive due to the different solvents used in their study.

### 3.4. Zeta-Potential Measurements

Al_2_O_3_, CeO_2_, and AlCeO_3_ nanoparticles with a final concentration of 0.1 g/L were prepared by diluting the nanopowder for each metal oxide in deionized water. Each suspension was then sonicated at 20 KHs at an intensity of 200 W/L for 15 min. The sample was then transferred immediately for zeta-potential measurement using the Zetasizer Nano ZS (Malvern) laser Doppler microelectrophoresis in an ac electric field.

**Figure 6:**
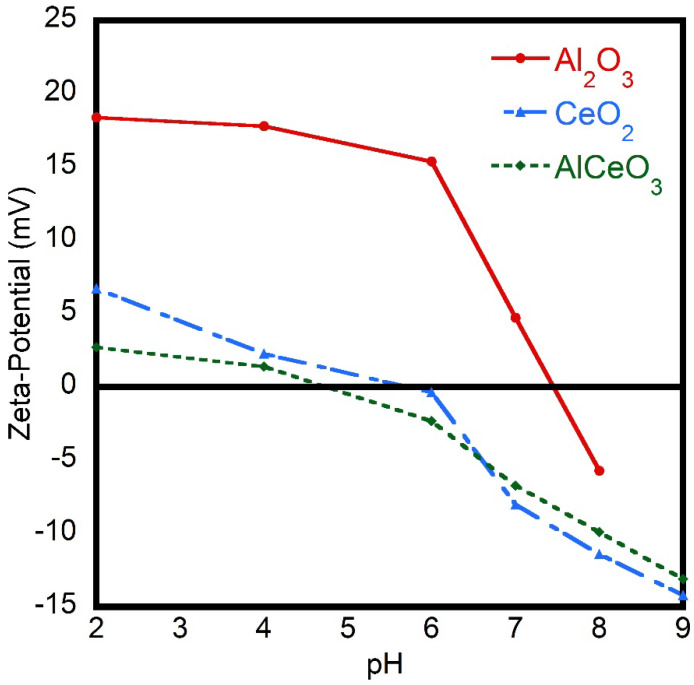
Zeta-potential vs. pH values of Al_2_O_3_, CeO_2_, and AlCeO_3_ nanopowders.

The zeta potential for Al_2_O_3_, CeO_2_, and AlCeO_3_ nanoparticles as a function of pH is demonstrated in Figure 6. The isoelectric point (iep) was observed at a pH of ≈7.5, ≈5.8, and ≈4.65 for Al_2_O_3_, CeO_2_, and AlCeO_3_, respectively. Authors have reported that that the isoelectric point for Al_2_O_3_ lies at pH 8, which is in agreement with the measured data [30]. It can be seen that the zeta potential is mostly positive in the acidic range. When the pH is less than 7, a large number of H^+^ ions are present due to the acidic nature of the nanoparticles. The increase in the positive zeta-potential values is justified by the increase in the presence of H+ ions in the electric double layer. On the other hand, a large number of OH- ions are present when the pH is larger than 7 due to the basic nature of the nanoparticles. For CeO_2_, the isoelectric value was obtained at 5.8, which is consistent with published measurements [31]. For acidic solutions, the net surface charge of the particles is less positive compared to AlCeO_3_ nanoparticles, whereas a negative charge is observed for alkaline solutions.

## 4. Conclusions

The study reported here evaluated the wettability of transition-metal oxide nanoparticles (Al_2_O_3_, CeO_2_, and AlCeO_3_). TEM and SEM results show the formation of nanopowders with a nanoparticle size of ≈10–80 nm. In addition, XRD powder diffraction patterns confirm the crystalline nature of the nanopowders prepared. The contact angles of water droplets on glass surfaces were measured as 77.5 ± 5°, 89.8 ± 4°, and 69.2 ± 1° for Al_2_O_3_, CeO_2_, and AlCeO_3_, respectively. The zeta-potential values varied with the nanoparticle constitution. The isoelectric point (iep) was observed at a pH of ≈7.9, ≈5.8, and ≈4.65 for Al_2_O_3_, CeO_2_, and AlCeO_3_, respectively. The contact angles for water were greatly affected by the size of nanoparticles. Furthermore, this study found a correlation between the contact angles and the chemical composition of nanoparticles, which can be investigated further in the future to systematically study the influence of chemical compositions on contact angles. The outcomes of this study can be incorporated in future studies related to the pharmacology for drug formulation and oil recovery from reservoir rocks fields.

## Data Availability

All datasets used for this study are included in the manuscript.
